# Antitumor activity of miR-34a in peritoneal mesothelioma relies on c-MET and AXL inhibition: persistent activation of ERK and AKT signaling as a possible cytoprotective mechanism

**DOI:** 10.1186/s13045-016-0387-6

**Published:** 2017-01-18

**Authors:** Rihan El Bezawy, Michelandrea De Cesare, Marzia Pennati, Marcello Deraco, Paolo Gandellini, Valentina Zuco, Nadia Zaffaroni

**Affiliations:** 1 Molecular Pharmacology Unit, Department of Experimental Oncology and Molecular Medicine, Fondazione IRCCS Istituto Nazionale dei Tumori, 20133 Milan, Italy; 2 Colon-Rectal Cancer Surgery Unit, Department of Surgery, Fondazione IRCCS Istituto Nazionale dei Tumori, 20133 Milan, Italy

**Keywords:** miR-34a, Diffuse malignant peritoneal mesothelioma, Receptor tyrosine kinases, AXL, c-MET

## Abstract

**Background:**

The value of microRNAs (miRNAs) as novel targets for cancer therapy is now widely recognized. However, no information is currently available on the expression/functional role of miRNAs in diffuse malignant peritoneal mesothelioma (DMPM), a rapidly lethal disease, poorly responsive to conventional treatments, for which the development of new therapeutic strategies is urgently needed. Here, we evaluated the expression and biological effects of miR-34a—one of the most widely deregulated miRNAs in cancer and for which a lipid-formulated mimic is already clinically available—in a large cohort of DMPM clinical samples and a unique collection of in house-developed preclinical models, with the aim to assess the potential of a miR-34a-based approach for disease treatment.

**Methods:**

miR-34a expression was determined by qRT-PCR in 45 DMPM and 7 normal peritoneum specimens as well as in 5 DMPM cell lines. Following transfection with miR-34a mimic, the effects on DMPM cell phenotype, in terms of proliferative potential, apoptotic rate, invasion ability, and cell cycle distribution, were assessed. In addition, three subcutaneous and orthotopic DMPM xenograft models were used to examine the effect of miR-34a on tumorigenicity. The expression of miRNA targets and the activation status of relevant pathways were investigated by western blot.

**Results:**

miR-34a was found to be down-regulated in DMPM clinical specimens and cell lines compared to normal peritoneal samples. miR-34a reconstitution in DMPM cells significantly inhibited proliferation and tumorigenicity, induced an apoptotic response, and declined invasion ability, mainly through the down-regulation of c-MET and AXL and the interference with the activation of downstream signaling. Interestingly, a persistent activation of ERK1/2 and AKT in miR-34a-reconstituted cells was found to counteract the antiproliferative and proapoptotic effects of miRNA, yet not affecting its anti-invasive activity.

**Conclusions:**

Our preclinical data showing impressive inhibitory effects induced by miR-34a on DMPM cell proliferation, invasion, and growth in immunodeficient mice strongly suggest the potential clinical utility of a miR-34a-replacement therapy for the treatment of such a still incurable disease. On the other hand, we provide the first evidence of a potential cytoprotective/resistance mechanism that may arise towards miRNA-based therapies through the persistent activation of RTK downstream signaling.

**Electronic supplementary material:**

The online version of this article (doi:10.1186/s13045-016-0387-6) contains supplementary material, which is available to authorized users.

## Background

Diffuse malignant peritoneal mesothelioma (DMPM) is an uncommon though locally aggressive tumor that develops from mesothelial cells lining the peritoneal cavity [1]. DMPM prognosis is dismal and standard therapy, including palliative surgery, systemic/intraperitoneal chemotherapy, and abdominal irradiation, showed to be ineffective, with a median survival of about 1 year [1]. Currently, the most effective treatment is a loco-regional approach combining aggressive cytoreductive surgery (CRS) with hyperthermic intraperitoneal chemotherapy (HIPEC), which significantly extended survival in selected series of patients [1]. However, for recurrent patients and for those who are not eligible to CRS+HIPEC, the prognosis remains severe due to the lack of alternative treatment options [2]. Considering the rarity of the disease and the unavailability of experimental models, the biology of DMPM is still largely unknown. It is anticipated that advances in the knowledge of the mechanisms responsible for the biological aggressiveness and the relative chemoresistance of DMPM will allow the identification of relevant targets for the development of novel therapeutic strategies.

MicroRNAs (miRNAs) are single-stranded endogenous evolutionary conserved, non-coding RNA molecules acting as post-transcriptional regulators of gene expression [3]. Deregulated miRNA expression and/or function have been observed in a variety of human solid and hematological tumors and have been causatively linked to the pathogenesis of cancer [4]. Depending on their expression levels, cellular context, and target functions, miRNAs can act as oncogenes or tumor suppressors [4] and may represent novel targets or tools for cancer therapy [5].

MiR-34a is one of the most widely studied miRNAs in cancer. Its expression has been found to be decreased in a variety of human tumors [5] due to DNA copy number loss or epigenetic silencing through aberrant CpG methylation [6, 7]. Results of studies where the expression of miR-34a was manipulated in human tumor experimental models clearly showed that the miRNA acts as a tumor suppressor by regulating highly relevant processes such as proliferation, cell cycle, apoptosis, invasion, and metastasis [8]. Reinforced expression of miR-34a has been found to positively modulate drug response in cancer cells [8]. In addition, a liposomal nanoparticle-formulated synthetic miR-34 (MRX34) recently entered a phase I clinical study for patients with different tumor types [9].

No information concerning the expression and/or the functional role of miRNAs in DMPM is currently available in the literature. Based on the knowledge that several receptor tyrosine kinases (RTKs) are validated targets of miR-34a [8] and our previous results indicating that activation of downstream RTK signaling, in terms of phosphorylation/overexpression of extracellular signal regulated kinase ½ (ERK1/2), AKT, and mTOR, is present in a considerable fraction of DMPM clinical specimens [10], we proposed to investigate the possible relevance of miR-34a in the disease, with the final aim to develop novel therapeutic strategies. Here, we report that miR-34a is down-regulated in DMPM clinical specimens and demonstrate that miR-34a replacement in a unique collection of in-house-developed human DMPM experimental models [11–13] inhibits cell proliferation and invasion and impairs tumor growth formation in SCID mice, mainly as a consequence of c-MET and AXL inhibition. These findings identified miR-34a-AXL and -c-MET axes as promising therapeutic targets for DMPM. Moreover, we provide evidence of persistent activation of ERK1/2 and AKT as a possible cytoprotective mechanism to RTK inhibition by miR-34a.

## Methods

### Clinical samples

Forty-five DMPM specimens classified as epitheliod (40), sarcomatoid (1), and biphasic (4) from patients treated with CRS+HIPEC at the Fondazione IRCCS Istituto Nazionale dei Tumori, Milan (INT) from October 1997 to February 2013, and 7 normal peritoneum specimens from patients who underwent surgery for non-oncologic disease were available for miR-34a expression analysis.

This study was approved by the Institutional Review Board and Ethical Committee and each patient provided written informed consent to donate to INT the leftover tissue after diagnostic and clinical procedures.

### Cell lines and culture conditions

The human mycoplasma-free DMPM cell lines MesoII, STO, MP115, MP4, and MP8 were established in our laboratory [11–13]. All cells were cultured in DMEM F-12 medium (Lonza, Milano s.r.l., Treviglio, Italy) supplemented with 10% fetal bovine serum in a 37 °C humidified 5% CO_2_ incubator. Cell lines were authenticated by single-tandem repeat analysis by the AmpFISTR Identifiler PCR amplification kit (Applied Biosystems, Foster City, CA, USA).

### Cell transfection

Mimic pre-miR-34a precursor (miR-34a) and mimic negative control (Neg) were purchased as Pre-miR™ miRNA precursor molecules (Thermo Fisher Scientific, Monza, Italy). Knockdown of AXL and c-MET was performed using specific siRNAs (siAXL and siMET; ON-TARGET plus SMART pool) and, as a control, a siRNA with a nonsense/scrambled sequence (siNeg, ON-TARGET plus non-Targeting Pool) (Dharmacon, CO, USA) was used. Cells were transfected for 24 h with 20 pM miR-34a or Neg, or 100 nM siAXL, siMET, or siNeg, using Lipofectamine® RNAiMAX Transfection Reagent (Thermo Fisher Scientific) with Opti-MEM I (Gibco, NY, USA) according to the manufacturer’s instructions.

### RNA extraction, cDNA synthesis, and qRT-PCR

Quantification of miR-34a expression levels was assessed by qRT-PCR. Total RNA was isolated using the miRNeasy Mini Kit (QIAGEN, Hilden, Germany) and 1 μg of RNA was reverse transcribed by miScript II RT Kit (QIAGEN). Mature miRNA expression was assayed by miScript Primer Assays specific for miR-34a (MS00003318) and normalized on SNORD48 (MS00007511) (QIAGEN). Quantitative RT-PCR was conducted using miScript SYBR Green PCR Kit (QIAGEN). The reaction was carried out in a 96-well PCR plate at 95 °C for 15 min followed by 40 cycles of 94 °C for 15 s, 55 °C for 30 s, and 70 °C for 30 s and a dissociation step to distinguish specific from non-specific amplification products. Each sample was analyzed in triplicate.

Amplifications were run on the 7900HT Fast Real-Time PCR System (Applied Biosystem). Data were analyzed by SDS 2.2.2 software (Applied Biosystems) and reported as -ΔCt, that is the difference between the Ct of the target gene and the Ct of the housekeeping gene (where Ct is the threshold cycle), or as relative quantity (RQ) or -ΔΔCt with respect to a calibrator sample (i.e., negative control transfected cells) according to the 2^−ΔΔCt^ method.

### Cell growth assay

To assess the effect of miR-34a restoration on cell proliferation, DMPM cells were transfected with Neg or miR-34a as described above. At different intervals from transfection, cells were trypsinized and counted in a particle counter (Beckman Coulter, Cassina de’ Pecchi, Italy). Results were expressed as percent variation in the number of miR-34a-transfected cells compared with Neg-transfected cells.

### Immunoblotting analyses

Cell lysates were fractionated by SDS-PAGE, transferred to nitrocellulose membranes, and probed with specific antibodies, as described in [14]. Cells were lysed and western blot was performed using the following primary antibodies: anti-c-MET, −CDK6, −uPA, −pospho-FAK (Tyr 576/577) (Santa Cruz Biotechnology, CA, USA); anti-AXL, −phospho-AKT (Ser473), −phospho-p44/42 MAPK (ERK1/2) (Thr202/Tyr204), −p44/42 MAPK (ERK1/2), −FAK, −cleaved CPP32 (Cell signaling, Beverly, USA); anti-AKT (BD Biosciences, San Jose, CA, USA); and anti-actin and -vinculin (Sigma Chemical Company, St. Louis, MO, USA).

Secondary antibodies used were conjugated to horseradish peroxidase (GE Healthcare, Little Chalfont, UK). Immunostained bands were detected by chemoluminescence method (ECL, GE Healthcare). In many experiments, membranes were stripped and reblotted with a second antibody. Moreover, membranes were cropped to allow simultaneous incubation of different primary antibodies on the same samples. For the preparation of figures, we cropped the original western blot to generate the appropriate figure panels with the relevant lanes. This cropped image was then subjected to uniform image enhancement of contrast and brightness. Molecular weights were determined using the Precision Plus Protein™ Standard (Bio-Rad, Segrate, Italy), which yields a colorimetric image only and has been removed from the chemoluminescent blot image.

### Drugs

The AKT-1/2 inhibitor trifluoroacetate salt hydrate (A6730, Sigma Chemical Company) and the MEK inhibitor CI-1040 (PD184352, Selleck Chemicals, Houston, TX, USA) were dissolved and diluted in DMSO. Final concentration of DMSO in cell cultures never exceeded 0.5%. The antiproliferative activity was evaluated by cell counting at different times after exposure of miR34a-reconstituted MesoII cells to drug concentrations able to inhibit cell proliferation by 20% (IC_20_).

### Apoptosis detection and cell cycle analysis

At different time points after transfection with miRNAs mimic or siRNAs, floating and adherent cells were harvested and processed for apoptosis evaluation by TUNEL assay according to manufacturer’s instructions (Roche, Mannheim, Germany) and for cell cycle [15]. For cell cycle, cells were fixed in 70% ethanol 96 h after transfection, stained in phosphate-buffered saline (PBS) containing 10 μg/ml propidium iodide (PI; Sigma Chemical Company), and RNase A (66 U/ml; Sigma Chemical Company) for 18 h and analyzed by FACScan flow cytometer (Becton Dickinson, Mountain View, CA, USA).

### Senescence-associated β-galactosidase staining

Cells were transfected with miR-34a or Neg for 24 h. Samples were washed in PBS 72 h after transfection and processed for senescence-associated β-galactosidase (SA-β-Gal) staining. Cells were fixed for 5 min (room temperature) in 2% formaldehyde/0.2% glutaraldehyde, washed and incubated overnight at 37 °C (no CO_2_) with fresh solution as previously described [15]. At least 300 cells were examined, and the results were expressed as percentage of SA-β-Gal positive cells over the whole population.

### Transwell invasion assay

Invasion assay was performed 72 h after transfection using a 24-well Boyden chamber with 8-mm pore size filter in the inset chambers (Costar, Corning Inc., NY, USA). The Transwell membranes were previously coated with 3.47 μg Matrigel/well (BD Biosciences) and dried for 30 min. Cells were suspended in 300 μL serum-free medium and seeded into the insert chambers. After 24 h of incubation at 37 °C in 5% CO_2_, cells that migrated into the bottom chamber containing 1 ml of serum-free medium were fixed in 95% ethanol, stained with a solution of 0.4% sulforhodamine B in 0.1% acetic acid, counted under an inverted microscope, and then photographed.

### Antibody arrays and ELISA

Cells were seeded at 2 × 10^4^ cells/dish in complete medium and transfected with Neg or miR-34a for 24 h before serum starvation for 72 h. Conditioned media were then harvested and clarified by centrifugation at 13,000 rpm for 15 min. Cells were trypsinized, counted, and lysed for assaying protein content. Supernatant aliquots were used to assess angiogenesis-related protein content by Antibody Arrays (R&D System, SPACE Import Export, Milan, Italy) according to manufacturer’s instructions. The ELISA kit for Maspin (human Maspin “Super X” ELISA Kit, Antigenix America, Huntington Station, NY, USA) was used according to the manufacturer’s instructions for quantitative analysis.

### In vivo experiments

All experimental protocols were approved by the Ethics Committee for Animal Experimentation of INT. Experiments were performed using 8-week-old female SCID mice (Charles River, Calco, Italy). Each group contained five to six mice. Cells were transfected with miR-34a or Neg for 24 h, as described above, and then inoculated subcutaneously or intraperitoneally after the analysis of the transfection efficiency by qRT-PCR.

#### Subcutaneous tumor models

STO, MesoII, and MP8 cells were injected subcutaneously into the right flank (1–1.2 × 10^7^ cells/mouse). Inoculated animals were inspected daily to establish the time of tumor onset. Tumor growth was measured every 2 to 3 days using a Vernier caliper (Table 1). The subcutaneous tumor volume was calculated as follows: TV (mm^3^) = *d* ^2^ × *D*/2 where *d* and *D* are the shortest and the longest diameter, respectively. Volume inhibition percentage (TVI%) in tumors derived from miR-34a- over Neg-transfected cells was calculated as follows: TVI% = 100 − (mean miR-34a TV/mean Neg TV × 100).

**Table 1:** Effect of miR-34a reconstitution on DMPM cell tumorigenicity following s.c. injection in SCID mice

Model	miRNA	Engrafted tumors/total mice^a^	Tumor onset^b^	TV (mm^3^)	TVI %^c^ (day)	*p* value^d^
STO	Neg	6/6	1	440 ± 94		
	miR-34a	6/6	1	188 ± 39	57 (10)	0.0003
MesoII	Neg	5/5	7	201 ± 93		
	miR-34a	5/5	12	9 ± 12	96 (12)	0.0035
MP8	Neg	5/5	18	72 ± 35		
	miR-34a	5/5	25	1 ± 2	98 (21)	0.0041

^a^Number of mice presenting s.c. tumors out of number of cell-injected mice

^b^Median day of tumor appearance

^c^Tumor volume inhibition % in miRNA34a- over Neg-transfected cell-injected mice

^d^By Student’s *t* test over Neg-transfected cell-injected mice

Proteins were obtained as described previously [16] from frozen s.c. tumors derived from two additional mice sacrified at different time points. Briefly, samples were pulverized by Mikro-Dismembrator II (B. Brown Biotech International, Melsungen, Germany) and suspended in lysis buffer supplemented with protease and phosphatase inhibitors. Proteins were processed as described [16].

#### Intraperitoneal (orthotopic) tumor models

STO and MP8 cells were injected into the peritoneal cavity (10^7^ and 2.5 × 10^7^ cells/mouse, respectively). Animals were monitored and weighed daily and sacrificed at different times from cell injection (Table 2). A careful necropsy was performed to evaluate the take rate and spread of mesothelioma cells in the abdominal cavity.

**Table 2:** Effect of miR-34a reconstitution on DMPM cell tumorigenicity following i.p. injection in SCID mice

Model	miRNA	Day of sacrifice	Engrafted tumors/total mice^a^	*p* value^b^	Tumor weight (mg)	Average value	TVI (% Neg)^c^	*p* value^d^
STO	Neg	14	5/5		10, 80, 80, 100, 110	76		
	miR-34a		1/5	0.0476	0, 0, 0, 0, 10	2	97	0.0029
MP8	Neg	31	5/5		130, 310,250, 210,150	210		
	miR-34a		5/5		80, 90, 70, 120, 80	88	58	0.0071

^a^Number of mice presenting i.p. tumors out of number of cell-injected mice

^b^By Fisher’s exact test over Neg cell-injected mice

^c^Tumor weight inhibition % in miRNA34a- over Neg-transfected cell-injected mice

^d^By Student’s *t* test over Neg-transfected cell-injected mice

Solid masses were gently detached from organs and abdominal walls, removed, and weighed for calculating the percentage of tumor weight inhibition (TWI %) in mice inoculated with miR-34a- over Neg-transfected cells.

### Statistical analyses

If not otherwise specified, in vitro data are presented as mean values ± SD from at least three independent experiments. Statistical analysis of the data was performed by two-tailed Student’s *t* test. For in vivo data, two-tailed Student’s *t* and Fisher’s exact test were used to compare tumor volumes/weights and tumor takes, respectively. Patient survival analysis was performed using Cox proportional regression model [17]. *p* values <0.05 were considered statistically significant.

## Results

### miR-34a is down-regulated in DMPM clinical samples and cell lines

**Fig. 1:**
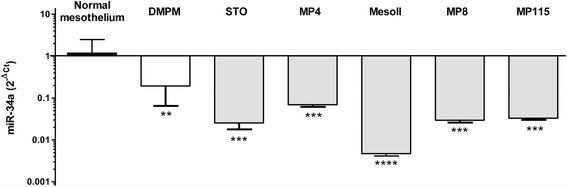
Expression levels of miR-34a. qRT-PCR analysis of miR-34a expression using total RNA from fresh normal peritoneum tissues (*n* = 7), DMPM clinical samples (*n* = 45), and DMPM cell lines (STO, MP4, MesoII, MP8, MP115). Data were presented as 2^−∆Ct^ ^(miR-34a-SNORD48)^ values (**p < 0.01; ***p < 0.001; ****p < 0.0001 by Student’s *t* test)

We first evaluated miR-34a expression by qRT-PCR in 45 DMPM and 7 normal peritoneum specimens as well as in 5 unique cell lines established in our laboratory from clinical samples of epithelioid (STO, MP4, MesoII, MP8) and biphasic (MP115) DMPM. Results indicated that miR-34a abundance is significantly reduced in DMPM compared to normal tissues (Fig. 1). Consistently, miR-34a expression was found down-regulated in all DMPM cell lines, thus indicating an oncosuppressive function of the miRNA also in this disease.

No significant difference in miR-34a expression was observed as a function of demographic and clinico-pathologic characteristics, including gender, histologic subtype, and peritoneal cancer index [18] (data not shown). In addition, at 5 years of follow-up, miR-34a expression did not significantly affect the probability of disease-free survival of DMPM patients (high expressing versus low expressing—categorized on the basis of the median miR-34a expression value—36 versus 20%; hazard ratio, 1.85; 95% confidence interval, 0.86–4.01; *p* = 0.11).

Overall, such findings suggest a role for miR-34a as a possible therapeutic target rather than a prognostic/predictive biomarker in DMPM.

### miR-34a reconstitution variably affects DMPM cell growth and apoptosis

**Fig. 2:**
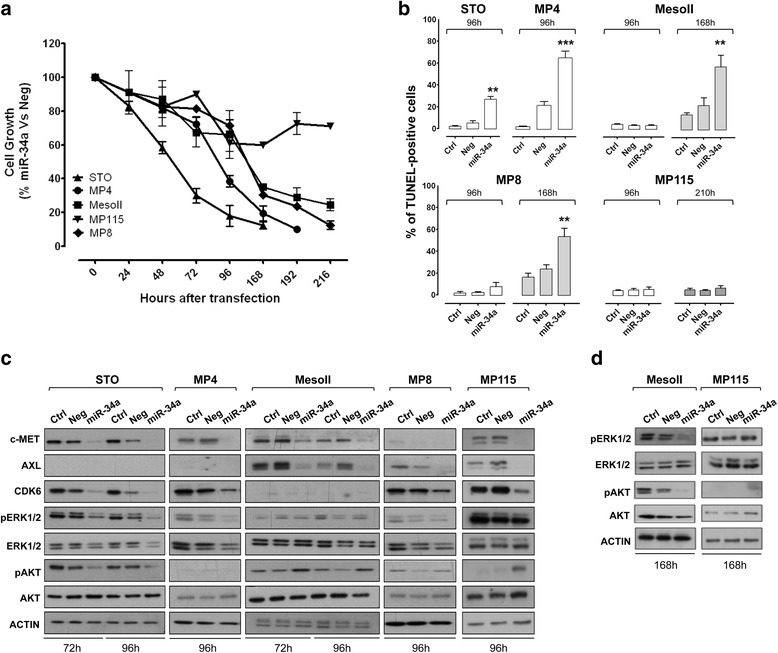
Effects of miR-34a reconstitution on DMPM cell proliferation, apoptosis, and RTK signaling pathways. **a** Antiproliferative effects of miR-34a. DMPM cell growth was assessed by cell counting. Data are expressed as percentage of the proliferation of miR-34a- versus miRNA negative control (Neg)-transfected cells. Means ± SD values of three independent experiments are reported. **b** Proapoptotic effects of miR-34a. Quantitative analysis of TUNEL-positive DMPM cells transfected with transfection reagent (*Ctrl*), Neg, or miR-34a was carried out by flow cytometry at different time points after transfection. Mean ± SD values of three independent experiments are reported (**p < 0.01; ***p < 0.001 by Student’s *t* test). **c**, **d** Effects of miR-34a on validated miRNA targets and RTK downstream signaling cascades as assessed by western blot analysis at 72, 96 (**c**) and 168 (**d**) h after cell transfection with Ctrl, Neg, or miR-34a. Actin was used to confirm equal protein loading. A representative experiment of three was reported. Cropped images of selected proteins are shown

To functionally assess the possible role of miR-34a as a novel therapeutic target in DMPM, we transiently transfected cells with miR-34a synthetic mimic and miRNA negative control. As assessed by qRT-PCR, marked increase in miRNA abundance was consistently observed in all cell lines at 24 h from transfection (Additional file 1: Figure S1) and was still maintained, although to a lesser extent, at 168 h (Additional file 1: Figure S1 and data not shown). miR-34a reconstitution significantly inhibited the proliferation of four out of five DMPM cell lines in a time-dependent manner, though with a different kinetics (Fig. 2a). Specifically, a more rapid cell growth decline was observed in STO and MP4 cells (~80% inhibition at 96 and 168 h, respectively), whereas, in MesoII and MP8 cells, the same level of inhibition was recorded at later time points (192 and 216 h, respectively) (Fig. 2a). Conversely, ectopic expression of miR-34a only induced a weak inhibition (~30%) of MP115 cell growth, which was almost constant until the end of the experiment (Fig. 2a).

The variable antiproliferative effects consequent to miR-34a reconstitution in the different DMPM cell lines was paralleled by a different kinetics of apoptosis induction, as detected by TUNEL assay (Fig. 2b). Specifically, a significant enhancement in the percentage of apoptotic cells was already appreciable at 96 h upon transfection of STO and MP4 cells, whereas the apoptotic response was induced at a later time point (168 h) in MP8 and MesoII cells (Fig. 2b). No induction of apoptotic cell death was observed in MP115 cells until 216 h after miR-34a reconstitution (Fig. 2b).

To investigate whether validated targets of miR-34a were modulated by its synthetic mimic in DMPM cells, we assessed protein expression levels of c-MET, AXL, and CDK6 considering their established role in the control of cell proliferation and apoptosis in different tumor types [8, 19–21]. A marked down-modulation of the three proteins was consistently observed in all DMPM cell lines (Fig. 2c), regardless of the effects induced by miR-34a reconstitution on cell growth and apoptosis.

### ERK1/2 and AKT activation as a possible cytoprotective mechanism following miR-34a reconstitution

Based on the evidence that the activation of downstream RTK signaling pathways, including PI3K/AKT and RAF/MEK/MAPK cascades, seems to be crucial in both malignant pleural [22] and peritoneal [10] mesothelioma, we evaluated the effect of miR-34a reconstitution on the phosphorylation status of AKT and ERK1/2 in DMPM cell lines. A reduced abundance of phospho-ERK1/2 and phospho-AKT was appreciable in STO cells at 72 and 96 h after transfection with miR-34a mimic (Fig. 2c). A reduced expression of phospho-ERK1/2 was also observed in miR-34a-reconstituted MP4 and MP8 cells at the latter time point (Fig. 2c). Consistent with the delayed antiproliferative and proapoptotic response following miR-34a reconstitution, a decline in the expression levels of phospho-ERK1/2 and phospho-AKT was observed in MesoII cells at only 168 h (Fig. 2c, d), whereas no decrease in the abundance of the two phopho-proteins was found in the less sensitive MP115 cell line at either time point (Fig. 2c, d).

**Fig. 3:**
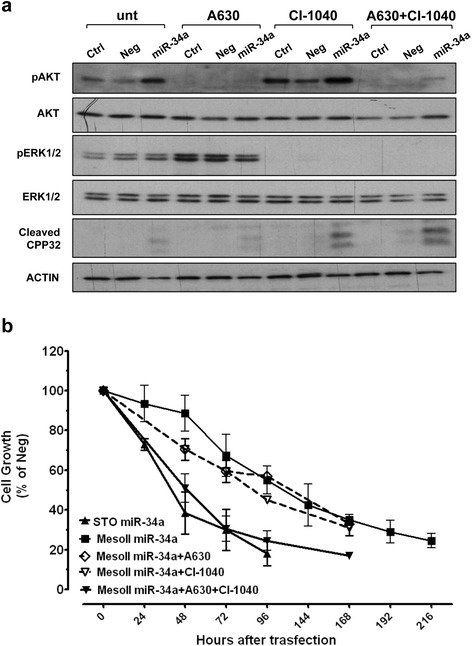
Inhibition of AKT and ERK1/2 signaling pathways increases the antiproliferative and proapoptotic effects of miR-34a. MesoII cells were transfected with Ctrl, Neg, or miR-34a for 24 h and successively exposed to vehicle (DMSO, unt) or low concentrations (corresponding to IC_20_ values) of A6730 (AKT inhibitor, 5 μM) and/or CI-1040 (MEK inhibitor, 3 μM). **a** Expression and phosphorylation status of AKT and ERK1/2 and amount of cleaved CPP32 at 72 h after drug treatment were assessed by western blot. Actin was used to confirm equal protein loading. A representative experiment of three was reported. The panel shows cropped blots. **b** The effects of AKT and MEK inhibitors on the growth of miR-34a-transfected cells were assessed by cell counting. Data are expressed as percentage of the proliferation of miR-34a- versus Neg-transfected cells. The cell growth inhibition curve of STO cells after enforced expression of miR-34a was reported for comparison. Means ± SD values of three independent experiments are shown

To assess whether activation of AKT or MAPK/ERK1/2 signaling pathways, which is a well-known mechanism of resistance to RTK inhibitors [23–26], could also represent a cytoprotective mechanism to the oncosuppessive effects of miR-34a, we exposed miRNA-reconstituted MesoII cells to subtoxic concentrations of small-molecule AKT (A6730) and MEK1 (CI-1040) inhibitors (Fig. 3a). Interestingly, inactivating ERK1/2 or impeding the reactivation of AKT only slightly affected MesoII response to miR-34a, whereas the concomitant blockade of the two pathways, made the sensitivity profile of MesoII cells superimposable on that of the inherently sensitive STO cell line (Fig. 3b). Such a growth inhibitory effect was paralleled by an earlier onset of apoptosis, as detected by caspase-3 cleavage (CPP32) at 96 h (Fig. 3a).

**Fig. 4:**
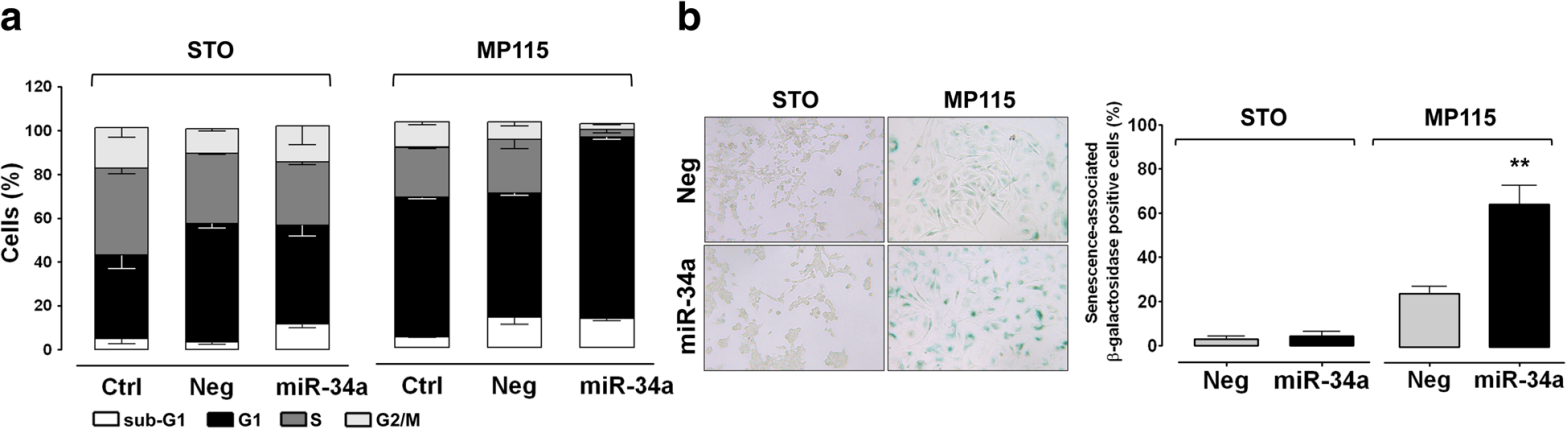
miR-34a induces a senescence-like phenotype in MP115 cells. **a** At 96 h following transfection of STO and MP115 cells with Ctrl, Neg, or miR-34a, nuclei were stained with propidium iodide and analyzed for DNA content by FACScan. Data (mean ± SD of three independent experiments) represent the percentage of cells in the different cell cycle phases. **b** *Left panel*: representative micrographs of SA-β-gal staining of STO and MP115 cells transfected with Neg or miR-34a. Blue precipitation in the cytoplasm was observed in the senescent cells. Original magnification, × 40. One representative experiment of three was shown. *Right panel*: *histogram bars* represent the mean percentage of SA-β-gal positive cells ± SD of at least three independent experiments (**p < 0.01 by Student’s *t* test)

To better characterize the cytostatic—rather than cytotoxic—effect observed in MP115 cells following miR-34a reconstitution, we assessed cell cycle distribution by flow cytometry. A cell accumulation in the G1-phase, which was paralleled by an enhanced fraction of senescence-associated β-galactosidase-positive cells, was observed (Fig. 4a, b). Such a senescence-like phenotype, which was not appreciable in the other DMPM cell models (Fig. 4b and data not shown), could be related to the marked phospho-AKT accumulation observed in miR-34a-reconstituted MP115 cells, according to previous evidence indicating that constitutively active AKT induces senescence in human endothelial cells and human fibroblasts [27, 28].

### miR-34a oncosuppressive activities mainly rely on c-MET and AXL inhibition

**Fig. 5:**
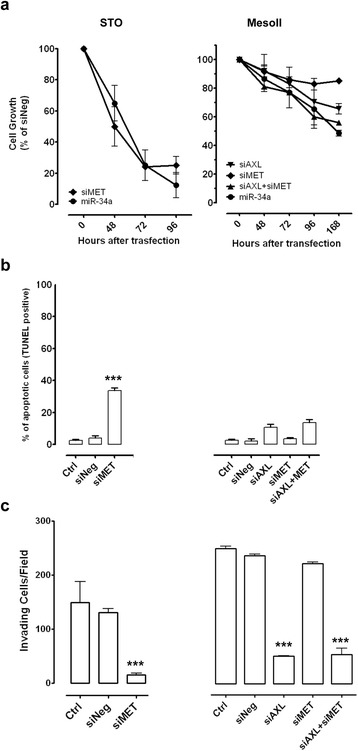
Silencing AXL and c-MET phenocopies miR-34a effects. STO and MesoII cells were treated with transfection reagent (Ctrl), siNeg (siRNA with a nonsense/scrambled sequence) or AXL- and c-MET-directed siRNA (siAXL, siMET) for 24 hours. **a** Effect of AXL and/or c-MET knockdown on cell growth, as detected by cell counting at different times after transfection. The antiproliferative effect induced by miR-34a reconstitution is reported for comparative purposes. **b** Induction of apoptosis at 96 h after transfection, as assessed by TUNEL assay (**p < 0.01 by Student’s *t* test). Data are expressed as percentage (mean ± SD) of the proliferation of siAXL-/siMET- versus siNeg-transfected cells. **c** Effect of RTK-siRNA on DMPM cell matrix degrading/invasive activities. Cells were silenced and, after 72 h, subjected to Matrigel invasion assay in serum-free medium. The number of invading cells per field is reported. *Histogram bars* represent mean values ± SD of at least three independent experiments (***p < 0.001 by Student’s *t* test)

To corroborate the hypothesis that miR-34a oncosuppressive functions mainly rely on the down-regulation of c-MET and AXL, we performed siRNA-based phenocopy experiments (Additional file 2: Figure S2). When transfected into STO cells, which do not inherently express AXL, siMET was able to recapitulate the effects induced by miR-34a reconstitution, in terms of cell growth inhibition (Fig. 5a), apoptosis induction (Fig. 5b), impairment of invasive capability (Fig. 5c), and inactivation of both ERK1/2 and AKT pathways (Additional file 2: Figure S2). In MesoII cells, siMET did not appreciably affect cell proliferation, apoptosis, or invasion (Fig. 5a–c). Conversely, siAXL reduced MesoII proliferation, although a cell growth inhibition comparable to that induced by miR-34a reconstitution was only observed following combined silencing of c-MET and AXL (Fig. 5a). Interestingly, siAXL alone phenocopied the effects of miR-34a, in terms of apoptosis and invasion (Fig. 5b, c), suggesting a main role of this RTK in mediating the oncosuppressive effects of miR-34a in MesoII cells. Moreover, AXL silencing did not inhibit AKT and ERK1/2 signaling pathways similarly to miR-34a reconstitution (Additional file 2: Figure S2).

The decreased EGFR abundance observed in miR-34a-reconstituted STO and MesoII cells (Additional file 3: Figure S3a), together with preliminary evidence indicating that the RTK is expressed/activated in DMPM clinical specimens [10], prompted us to investigate a possible role for EGFR down-regulation in sustaining the miR-34a-induced cell phenotype. However, siEGFR failed to affect cell growth and invasion capability in both cell lines (Additional file 3: Figure S3b).

### miR-34a reconstitution inhibits DMPM cell invasion and impairs the secretion of angiogenesis-related factors

**Fig. 6:**
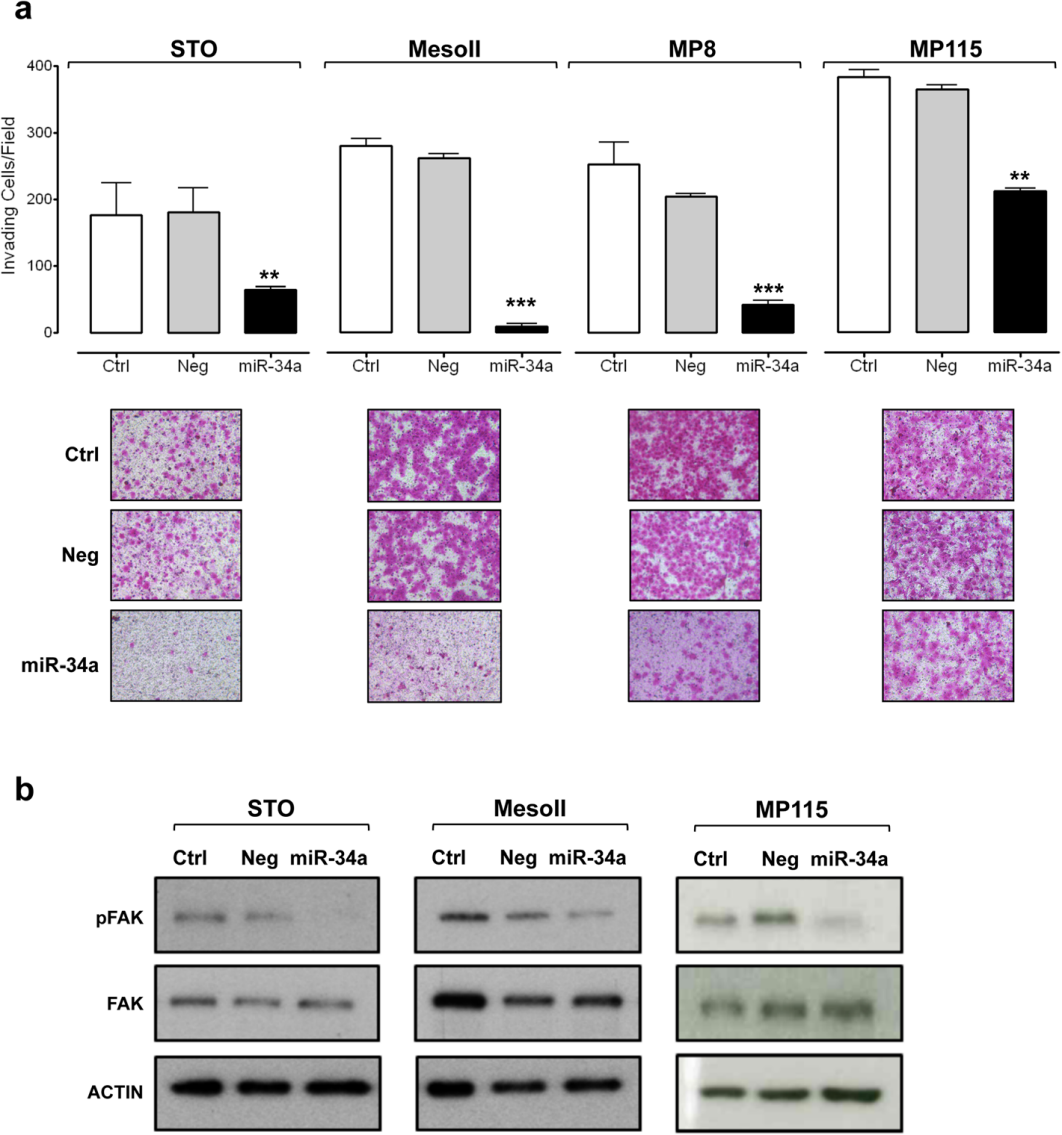
miR-34a inhibits the invasion of DMPM cells. Cells were transfected with Ctrl, Neg, or miR-34a for 24 h. **a** Cells were subjected to Matrigel invasion assay in serum-free medium 72 h after transfection. *Top*: the number of invading cells per field is reported. *Histogram bars* represent mean values ± SD of three independent experiments (**p < 0.01; ***p < 0.001 by Student’s *t* test). *Bottom*: micrographs from one experiment representative of three. Original magnification, × 40. **b** Whole-cell lysates were analyzed by western blot with anti-phospho-FAK. Protein extraction was performed 96 h after transfection with Ctrl, Neg, or miR-34a. Vinculin was used to confirm equal protein loading. A representative experiment of three was reported. Cropped blots are shown

Enforced expression of miR-34a significantly inhibited invasion of all DMPM cell models, as detected in a matrigel-based assay at 72 h after transfection (Fig. 6a), likely as a consequence of c-MET or AXL down-regulation. c-MET and AXL signaling pathways are indeed known to affect cell motility and invasion primarily through the activation of mitogen-activated protein kinase (MAPK) [29–32]. However, since a decreased invasive potential was observed also in MesoII and MP115 cells in spite of MAPK iperactivation (Fig. 2c), the status of focal adhesion kinase (FAK), known to mediate cell migration and anchorage-independent growth downstream of RTKs [29–33], was assessed. Interestingly, miR-34a ectopic expression consistently reduced FAK posphorylation at Y576/577 in STO, MesoII, and MP115 cell models, suggesting that miR-34a-induced inhibition of cell invasion can occur regardless of MAPK and AKT activation (Fig. 6b).

**Fig. 7:**
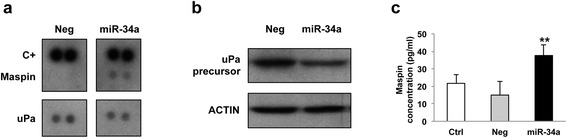
Effect of miR-34a restoration on the secretion of angiogenesis-related factors. **a** Secreted angiogenesis-related molecules were examined by antibody arrays in conditioned medium obtained from Neg- or miR-34a-transfected MesoII cells grown for 72 h in the absence of serum. C+, internal standards. The same conditioned media were used to assess the expression of **b** uPa precursor by western blot and **c** maspin protein by ELISA. Each bar represents mean ± SD of triplicate samples from a representative experiment (**p < 0.01 by Student’s *t* test)

Since AXL and c-MET inhibition by drugs and monoclonal antibodies was shown to induce antiangiogenic effects in tumors [34, 35], we investigated whether miR-34a reconstitution was able to affect the production/release of angiogenesis-related proteins by DMPM cells. We found that miR-34a reconstitution in MesoII cells impaired the secretion of angiogenesis-related molecules (Fig. 7). Indeed, antibody array results obtained in conditioned medium of miR-34a-reconstituted cells (at 72 h following transfection) showed a reduced secretion of the urokinase-type plasminogen activator (uPA), which plays a major role in promoting angiogenesis [36], together with and increased release of maspin, a member of the serine protease inhibitor (serpin) superfamily, which exerts antiangiogenic effects through the inhibition of both the growth and migration of endothelial cells [37, 38] (Fig. 7a). Western blot and ELISA experiments carried out on the same conditioned media confirmed a reduced expression of uPA precursor (Fig. 7b) and an increased abundance of maspin (Fig. 7c).

### miR-34a reconstitution inhibits tumor formation in SCID mice

**Fig. 8:**
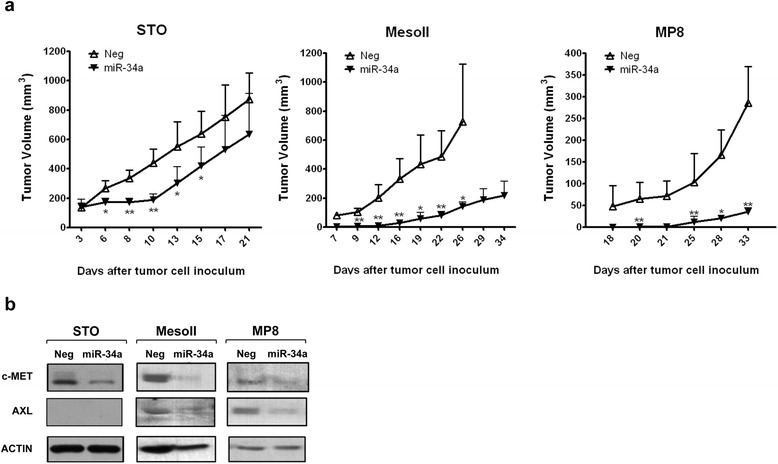
miR-34a inhibits DMPM tumorigenicity. **a** STO, MesoII, and MP8 cells were transfected with miR-34a or Neg and, on day 0, implanted subcutaneously into the right flank of SCID mice. Micrographs show the mean tumor volumes ± SD measured at different time points after DMPM cell injection. (*p < 0.05, **p < 0.01 by two-tailed Student’s *t* test). **b** Cropped western blot for c-MET and AXL expression in frozen tumors derived from two additional mice sacrificed at different times (STO, 10 days; MesoII, 19 days; MP8, 25 days) after cell inoculum. Actin was used to confirm equal protein loading

To investigate whether miR-34a reconstitution affected DMPM formation in vivo, we subcutaneously (STO, MesoII, MP8) and intraperitoneally (STO, MP8) inoculated miR-34a mimic- or miRNA negative control-transfected cells into SCID mice. Results indicate that miR-34a consistently impaired the growth of all s.c. xenograft models, with maximum tumor volume inhibitions ranging from 57 to 98% (Fig. 8 and Table 1). In addition, an appreciably delayed tumor onset was observed for MesoII and MP8 cell models (Table 1). Western blot carried out in tumors collected from additional mice sacrificed at different time points after DMPM cell inoculum indicated a decreased expression of c-MET in all xenograft models and AXL in MesoII and MP8 models (Fig. 8b), in accordance with in vitro results.

As regards i.p. xenograft models, at necropsy, all control mice showed a large tumor mass at the site of cell injection mainly invading the peritoneum wall and widespread small nodules in the peritoneum and attached to the diaphragm, liver, and bowel. Conversely, only one mouse out of the five mice receiving miR-34a-reconstituted STO cells developed small tumor nodules in the abdominal cavity (Table 2). miR-34a ectopic expression did not influence the take of MP8 cells but markedly reduced their growth, as indicated by a significantly reduced tumor weight (Table 2).

## Discussion

No information is currently available on the expression and functional role of miRNAs in DMPM. Here, we demonstrated that miR-34a is down-regulated in a large series of DMPM clinical samples and in a unique panel of cell lines, established from DMPM patients in our laboratories, compared to normal peritoneum specimens. We also illustrated that miR-34a exerts oncosuppressive functions in our tumor models, consistent to what previously observed in a variety of human tumor types [5, 39–41]. Indeed, miR-34a reconstitution impaired proliferation and induced an apoptotic response in DMPM cell lines, although at a variable extent and with different kinetics, mainly through the down-regulation of c-MET and AXL and the interference with the activation of downstream signaling. Interestingly, results also indicated that a transient or persistent activation of ERK1/2 and AKT can delay or prevent the cytotoxic and proapoptotic effects of miR-34a reconstitution, as observed in MesoII and MP115 cells, respectively. Noteworthy, DMPM cell feedback to AXL and c-MET down-regulation induced by miR-34a reconstitution is to directly activate ERK1/2 and AKT survival signaling cascades rather than up-regulate the expression levels of the receptors, thus ensuring a more prompt counter-response. Such findings provide the first evidence that tumor cells can exploit a well-known mechanism of resistance to RTK inhibitors—i.e., the activation of RTK downstream signaling [23–26]—to counteract the antiproliferative/proapoptotic effects of miR-34a. However, such a mechanism was not found to protect DMPM cells from the anti-invasive effect of the miRNA.

Noteworthy, the cytoprotective mechanism based on ERK1/2 and AKT activation was mainly evident in the DMPM cell line MP115, derived from a biphasic subtype tumor. Such subtype is known to be more aggressive and associated with a reduced patient survival compared to the epithelioid [42], although differences in specific relevant biological properties between the two DMPM subtypes are currently unknown. In addition, the delayed pro-apoptotic and cytotoxic effects observed in MesoII cells following miR-34a reconstitution are consistent with the finding that, unlike other epithelioid cell lines, they carry a mutant p53 [11].

A novel mechanism of miR-34a-dependent AKT inhibition has been recently proposed by Wang et al. [43]. In this study, miR-34a is reported to inhibit Bmi-1 by targeting c-Myc in gastric cancer cells, resulting in a PTEN-dependent reduction of phospho-AKT. The observation that in DMPM cell lines more susceptible to the cytotoxic effects of miR-34a (STO, MP4, MP8), a decrease in phospho-AKT abundance is observed early after miRNA reconstitution would suggest the possibility that the above-described mechanism is also operating in our models. However, results of phenocopy experiments showing that siRNA-mediated silencing of c-MET and AXL was able to decrease AKT activation in sensitive cells (STO) but not in those less susceptible to miR-34a (MesoII) would suggest that the main mechanism controlling AKT phosphorylation status relies on RTK activity.

Interestingly, miR-34a induced a remarkable antitumor activity in the three cell lines (STO, MesoII and MP8) able to generate tumors following xenotransplantation into immunodeficient mice. Although to a different extent, miR-34a reconstitution significantly reduced the growth of the three s.c. xenograft models. Highly relevant to the disease, miRNA ectopic expression also impaired the growth of STO and MP8 orthotopic xenografts, which properly recapitulate the dissemination pattern in the peritoneal cavity of human DMPM [11, 12], thus representing improved models to investigate novel therapeutic approaches. Specifically, miR-34a significantly inhibited the take of STO cells, with only one mouse developing small tumor nodules in the abdominal cavity. Although the miRNA did not influence the take of MP8 cells, a significantly reduced tumor growth was observed.

Unfortunately, the inability of MP115—the only biphasic DMPM model in our panel—to grow in vivo prevented us to assess whether the in vitro cytostatic effect consequent to miR-34a reconstitution, which was paralleled by the induction of a senescence-like phenotype possibly sustained by ATK activation, may result or not in tumor growth impairment. However, the significantly reduced invasive potential induced by miR-34a in DMPM cell lines through the inhibition of FAK signaling could primarily contribute to the antitumor effect observed in the xenograft models. Moreover, the occurrence of miR-34a-induced inhibition of cell invasion in the absence of appreciable antiproliferative and proapoptotic effects that we observed in MP115 is not surprising since the same phenotype has been previously reported by Li et al. [44] for miR-34a reconstituted HepG2 hepatocellular carcinoma cells.

Interestingly, our evidence indicating that miR-34a ectopic expression impairs the secretion of angiogenesis-related factors by MesoII cells strongly suggests that the antitumor effect observed in both s.c. and ortothopic xenograft models can also rely on miRNA-induced modification of tumor microenvironment, making it less favorable to tumor growth.

In summary, the impressive inhibitory effects induced by miR-34a on DMPM cell proliferation, invasion, and growth in immunodeficient mice suggest a possible utility of the clinically available miR-34a as novel therapeutic option for DMPM patients who are not eligible for or relapse after CRS+HIPEC. In addition, the evidence that miR-34a reconstitution positively modulates the activity of antitumor drugs in experimental models of different human tumor types [8, 45–47] highlights the possibility that the miR-34a mimic could have an important role also in combined strategies for treating DMPM patients.

## Conclusions

DMPM is a rapidly fatal tumor with scanty therapeutic options. Here, we demonstrated for the first time that reconstitution of miR-34a in relevant models of the disease induced a significant antitumor effect, which mainly relied on c-MET and AXL down-regulation and impairment of their downstream signaling. In vivo results were complemented by in vitro data showing significant antiproliferative, proapoptotic, and anti-invasive activities. Taken together, our results provide evidence that (i) c-MET and AXL signaling pathways are critical determinants of DMPM cell survival, growth, and invasiveness and that miR-34a reconstitution can impair all these functions and (ii) persistent activation of AKT and ERK1/2 downstream signaling pathways represents a cytoprotective mechanism against miRNA-induced proapoptotic effects, though not preventing its anti-invasive activity, which instead mainly relies on FAK inhibition.

Overall, our preclinical data form a solid foundation that could promote the clinical translation of clinically available miR-34 mimic for the treatment of a still incurable disease such as DMPM and, on the other hand, provide the first evidence of a possible cytoprotective/resistance mechanism that may arise towards miRNA-based therapeutics.

## Additional files

Additional file 1: Figure S1. Expression of miR-34a upon restoration in DMPM cells. Cells were transfected with either Neg or miR-34a for 24 h. (A) qRT-PCR analysis in the panel of DMPM cell lines 24 h after transfection. (B) miR-34a expression by qRT-PCR in MesoII at different times after transfection. Data are reported as ^−∆∆Ct^ between miR-34a- and Neg-transfected cells. A representative experiment of three was reported. (TIF 236 kb)
Additional file 2: Figure S2. Effects of silencing AXL and c-MET on RTK and downstream signaling pathways. DMPM cells were lysed 72 h after transfection with RNAimax (Ctrl), control siRNA (siNeg), or AXL- and c-MET-directed siRNAs (siAXL, siMET) for 24 h. RTK levels and activation status of ERK 1/2 or AKT were assessed by western blot analysis. Cropped images of the protein expression are reported. Vinculin was used to confirm equal protein loading. (TIF 559 kb)
Additional file 3: Figure S3. a and b Effect of EGFR modulation by miR-34a or siEGFR on DMPM cells. (a) EGFR protein expression was assessed by western blot analysis at 72 and 96 h after 24 h transfection with transfection reagent (Ctrl), Neg or miR-34a. Actin was used to confirm equal protein loading. Cropped blots are presented. (b) EGFR protein expression was determined by western blot analysis 72 h after 24 h transfection with siNeg or EGFR-directed siRNA (siEGFR). Vinculin was used to confirm equal protein loading. (c) DMPM cell proliferation was assessed by cell counting at different time points after 24 h transfection with siNeg or siEGFR. (d) DMPM cell invasion was determined in a Matrigel-based assay at 72 h after 24 h transfection with transfection reagent (Ctrl), siNeg, or siEGFR. (TIF 606 kb)

## Abbreviations

DMPM: Diffuse malignant peritoneal mesothelioma
ERK1/2: Extracellular signal regulated kinase ½
FAK: Focal adhesion kinase
MAPK: Mitogen-activated protein kinase
miRNA: MicroRNA
PI3K: Phosphoinositide 3-kinase
RTK: Receptor tyrosine kinase
TVI: Tumor volume inhibition
TWI: Tumor weight inhibition
uPA: Urokinase-type plasminogen activator
