# Supplementary figures and images for: Antitumor activity of miR-34a in peritoneal mesothelioma relies on c-MET and AXL inhibition: persistent activation of ERK and AKT signaling as a possible cytoprotective mechanism
Source: J Hematol Oncol. 2017 Jan 18;10:19. doi: 10.1186/s13045-016-0387-6 (PMC5242015; doi:10.1186/s13045-016-0387-6)

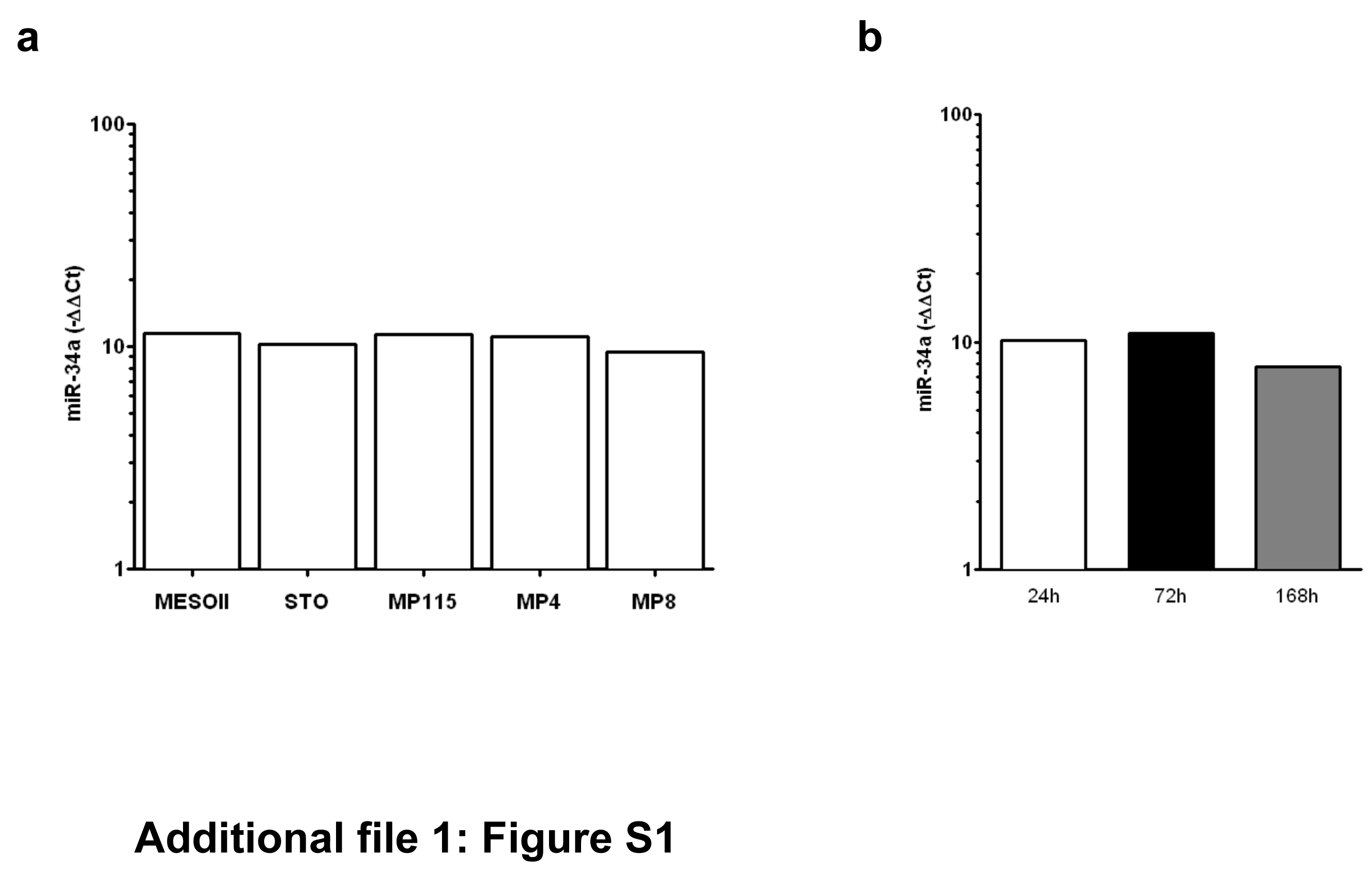

Supplement: Additional file 1: Figure S1. — Expression of miR-34a upon restoration in DMPM cells. Cells were transfected with either Neg or miR-34a for 24 h. (A) qRT-PCR analysis in the panel of DMPM cell lines 24 h after transfection. (B) miR-34a expression by qRT-PCR in MesoII at different times after transfection. Data are reported as −∆∆Ct between miR-34a- and Neg-transfected cells. A representative experiment of three was reported. (TIF 236 kb) [file 13045_2016_387_MOESM1_ESM.tif]

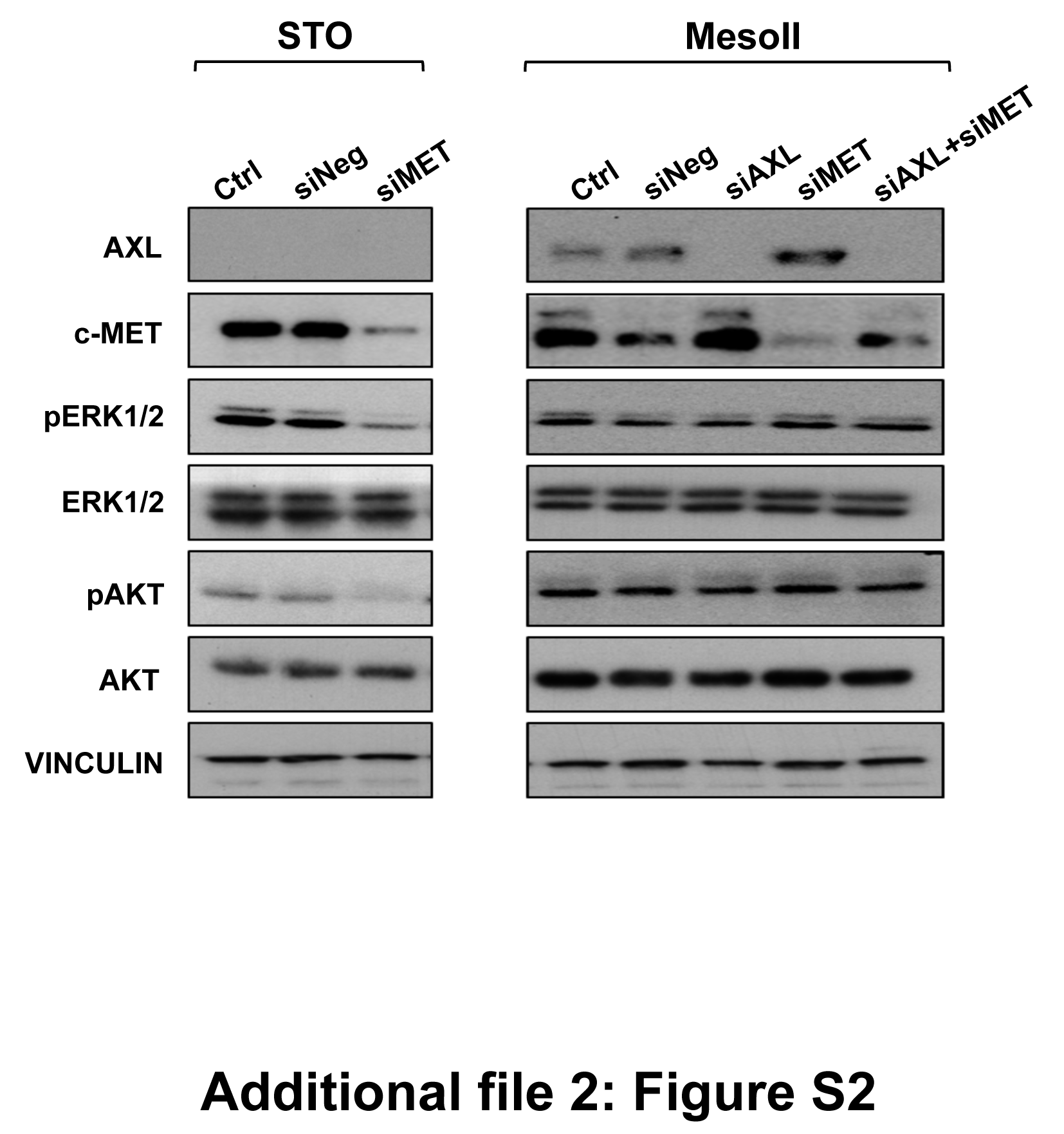

Supplement: Additional file 2: Figure S2. — Effects of silencing AXL and c-MET on RTK and downstream signaling pathways. DMPM cells were lysed 72 h after transfection with RNAimax (Ctrl), control siRNA (siNeg), or AXL- and c-MET-directed siRNAs (siAXL, siMET) for 24 h. RTK levels and activation status of ERK 1/2 or AKT were assessed by western blot analysis. Cropped images of the protein expression are reported. Vinculin was used to confirm equal protein loading. (TIF 559 kb) [file 13045_2016_387_MOESM2_ESM.tif]

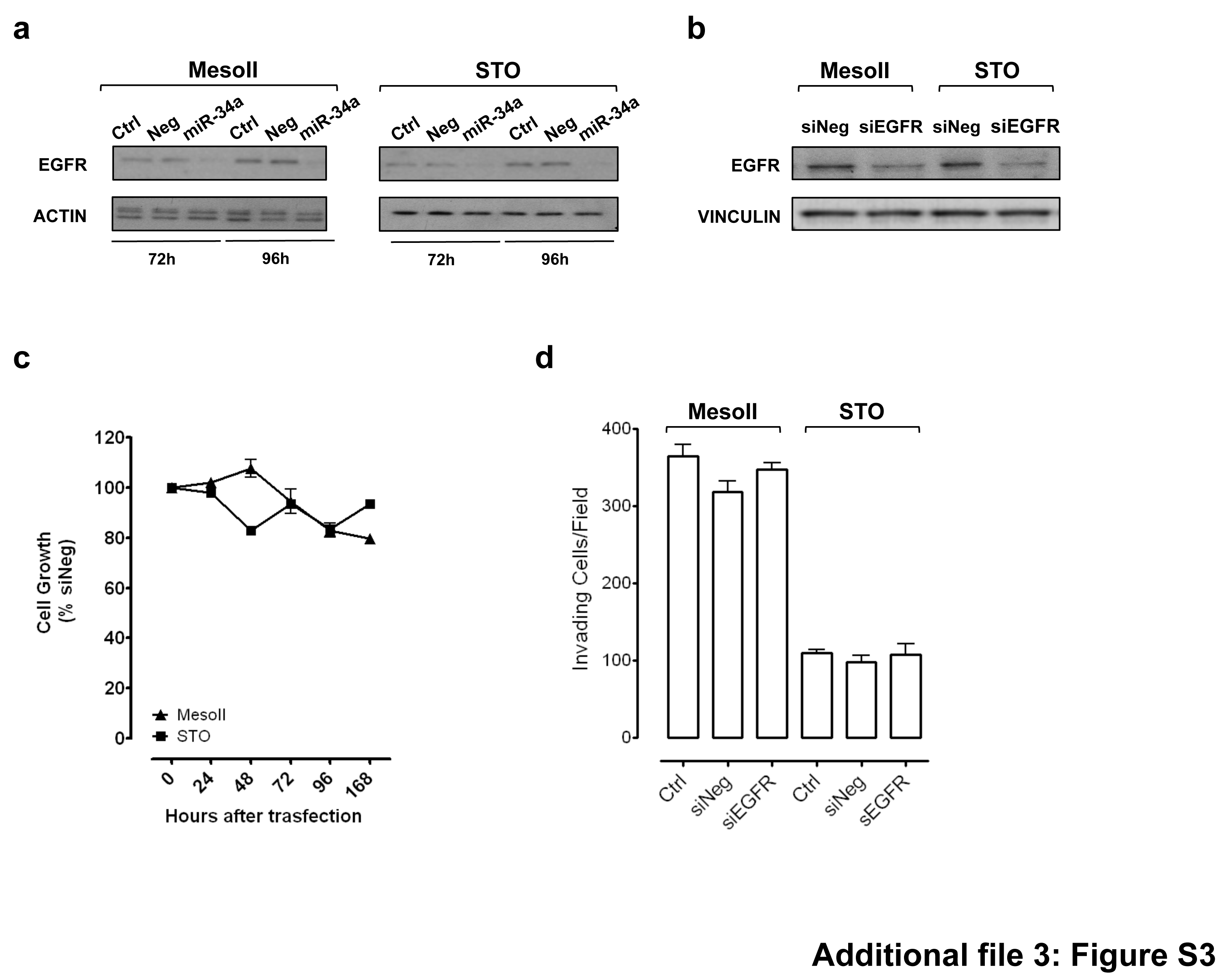

Supplement: Additional file 3: Figure S3. — a and b Effect of EGFR modulation by miR-34a or siEGFR on DMPM cells. (a) EGFR protein expression was assessed by western blot analysis at 72 and 96 h after 24 h transfection with transfection reagent (Ctrl), Neg or miR-34a. Actin was used to confirm equal protein loading. Cropped blots are presented. (b) EGFR protein expression was determined by western blot analysis 72 h after 24 h transfection with siNeg or EGFR-directed siRNA (siEGFR). Vinculin was used to confirm equal protein loading. (c) DMPM cell proliferation was assessed by cell counting at different time points after 24 h transfection with siNeg or siEGFR. (d) DMPM cell invasion was determined in a Matrigel-based assay at 72 h after 24 h transfection with transfection reagent (Ctrl), siNeg, or siEGFR. (TIF 606 kb) [file 13045_2016_387_MOESM3_ESM.tif]
